# Genome-Wide and Cell-Specific Epigenetic Analysis Challenges the Role of Polycomb in *Drosophila* Spermatogenesis

**DOI:** 10.1371/journal.pgen.1003842

**Published:** 2013-10-17

**Authors:** Sherif El-Sharnouby, Juliet Redhouse, Robert A. H. White

**Affiliations:** Department of Physiology, Development and Neuroscience, University of Cambridge, Cambridge, United Kingdom; Centre National de la Recherche Scientifique, France

## Abstract

The *Drosophila* spermatogenesis cell differentiation pathway involves the activation of a large set of genes in primary spermatocytes. Most of these genes are activated by testis-specific TATA-binding protein associated factors (tTAFs). In the current model for the activation mechanism, Polycomb plays a key role silencing these genes in the germline precursors, and tTAF-dependent activation in primary spermatocytes involves the displacement of Polycomb from gene promoters. We investigated the genome-wide binding of Polycomb in wild type and tTAF mutant testes. According to the model we expected to see a clear enhancement in Polycomb binding at tTAF-dependent spermatogenesis genes in tTAF mutant testes. However, we find little evidence for such an enhancement in tTAF mutant testes compared to wild type. To avoid problems arising from cellular heterogeneity in whole testis analysis, we further tested the model by analysing Polycomb binding in purified germline precursors, representing cells before tTAF-dependent gene activation. Although we find Polycomb associated with its canonical targets, we find little or no evidence of Polycomb at spermatogenesis genes. The lack of Polycomb at tTAF-dependent spermatogenesis genes in precursor cells argues against a model where Polycomb displacement is the mechanism of spermatogenesis gene activation.

## Author Summary

How are developmental gene expression programs activated? An interesting activation mechanism has been suggested by studies of the switch-on of the genes required for sperm differentiation in *Drosophila*; the spermatogenesis genes. These studies indicated that removal of the Polycomb protein from spermatogenesis genes was a key event in their activation. This was interesting because Polycomb is a central component of a gene regulatory complex that works at the level of chromatin and is best known for its role in stable heritable gene silencing. We have further analysed this system by examining Polycomb binding at the genome-wide scale and specifically in the precursor cells where Polycomb is proposed to repress the spermatogenesis genes. Surprisingly, this cell-specific analysis found no clear evidence for Polycomb binding at the spermatogenesis genes before their activation. This argues against the current model where Polycomb removal is the key mechanism involved in the activation of these genes. Further work is needed to identify the actual mechanism of gene activation in this system; however, our results fit with previous work suggesting that lamin-association rather than Polycomb binding may be responsible for repression of spermatogenesis genes prior to their activation.

## Introduction

The *Drosophila* spermatogenesis cell differentiation pathway involves the activation of over 1,000 genes in a testis-specific transcription program in primary spermatocytes. This represents a key developmental switch and provides an important model system for the analysis of regulatory mechanisms governing cell fate specification and pathway-specific gene expression programs (reviewed in [1]). Genetic screens and biochemical studies have led to the characterisation of at least two protein complexes responsible for the switch-on of this spermatogenesis transcription program. The first is a component of the core transcription machinery: a testis-specific TF_II_D complex containing testis-specific TATA-binding protein associated factors (tTAFs) [2], [3]. The second is the testis meiotic arrest complex (tMAC): a testis-specific version of the ubiquitous dREAM complex [4]–[9]. The dREAM complex was initially associated with repression but more recently has also been implicated in activation [10]–[13]. Both tTAFs and tMAC control similar sets of genes, although certain cell cycle genes involved in the G2/M transition of meiosis I appear to be dependent only on tMAC [1]. In addition, the two homeodomain transcription factors Achi (FBgn0033749) and Vis (FBgn0033748) are also required for spermatogenesis gene activation [14], [15].

How tTAFs and tMAC interact to switch on the primary spermatocyte transcription program is unclear, however the current model of spermatogenesis gene activation [16] indicates a key role for an interaction between tTAFs and Polycomb (Pc; FBgn0003042), a component of the Polycomb-group (PcG) silencing machinery (reviewed in [17]). It is proposed that the spermatogenesis genes are kept silent in the precursors (germline stem cells and spermatogonia) by Pc-mediated silencing. Then, in primary spermatocytes, the expression of tTAFs leads to a displacement of Pc from the promoters of spermatogenesis genes resulting in gene activation. In addition, loss of silencing appears to be aided by a tTAF-dependent sequestration of Pc to a compartment within the nucleolus [16], [18]. The key evidence for this model is that Pc binding to spermatogenesis gene promoters, demonstrated by chromatin immunoprecipitation (ChIP), is enhanced in tTAF mutant testes. This suggests that the wild type function of tTAFs is to displace Pc from the promoters of spermatogenesis genes, and that in tTAF mutants Pc silencing persists into the primary spermatocyte stage and inhibits the switch-on of the primary spermatocyte transcription program. This model is important as it provides a clear view of a cell differentiation switch mediated by removal of a component of the epigenetic PcG silencing machinery.

As this model was based on the analysis of Pc binding at only a few selected spermatogenesis gene promoters, we decided to extend the investigation to a genomic scale and examine the genome-wide binding of Pc in wild type and tTAF mutant testes. Contrary to what one would expect from the current model, our results show little evidence for enhanced Pc binding at tTAF-dependent spermatogenesis gene promoters in tTAF mutant testes. Considering that cellular heterogeneity may complicate whole testis analysis, we further investigated Pc binding in purified germline precursors, where we find no clear evidence for the binding of Pc at tTAF-dependent spermatogenesis genes. This argues that Pc displacement is not the mechanism involved in tTAF-dependent gene activation, challenging the existing model and highlighting the benefit of performing genome-wide epigenetic analysis in a cell type-specific manner.

## Results

### Pc binding in wild type and tTAF mutant whole testes

**Figure 1:**
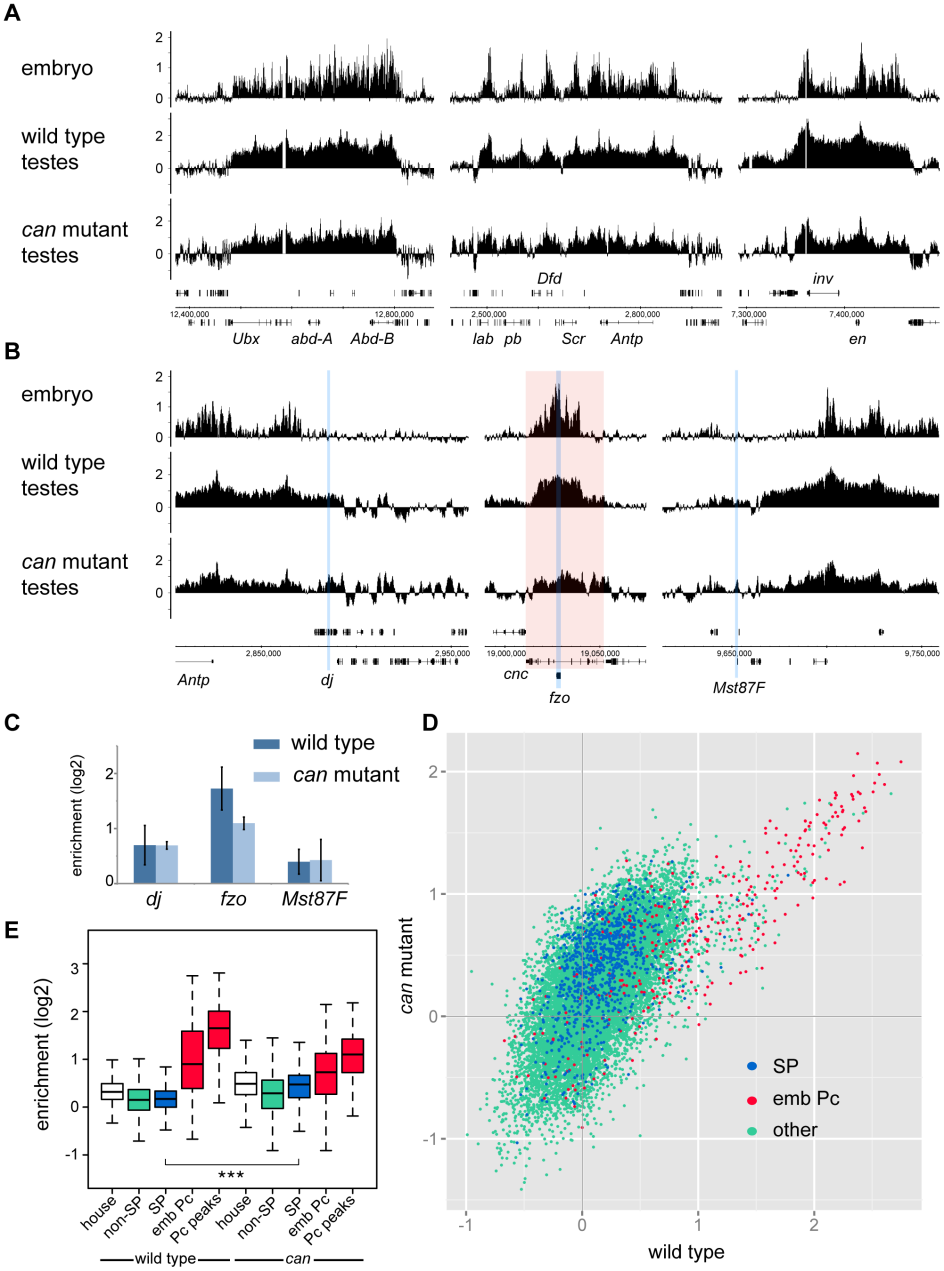
Pc binding in whole testes. (A,B) Log_2_ ChIP enrichment profiles for Pc in wild type and *can* mutant whole testes, with embryo data [19] shown for comparison. Profiles are shown for the canonical Pc targets of the bithorax complex, the Antennapedia complex and the *en* locus (A), and for the three tTAF-dependent spermatogenesis genes *dj*, *fzo*, and *Mst87F* (B; blue vertical stripes, red shading indicates the extent of the *cnc* gene). Linear scaled ChIP enrichment profiles are presented in Figure S1. (C) Average log_2_ ChIP enrichment for Pc over a 1 kb window at the TSS for the three spermatogenesis genes in wild type and *can* mutant testes (error bars = 1 SD, for wild type n = 3, for *can* mutant n = 2). (D) Scatterplot showing average log_2_ ChIP enrichment for Pc over a 1 kb window at the TSS for all genes in the genome in wild type versus *can* mutant testes; tTAF-dependent spermatogenesis genes (SP, blue), embryo Pc targets (emb Pc, red) and remaining genes (other, green). (E) Box plots showing the distribution of TSS Pc enrichments for defined gene sets in wild type and *can* mutant testes; tTAF-dependent spermatogenesis genes (SP, blue), embryo Pc targets (emb Pc, red), top embryo Pc peaks (Pc peaks, red), non-spermatogenesis genes (non-SP, green), and housekeeping genes (house, white). Boxplots show median, 25^th^ and 75^th^ percentiles and 1.5× interquartile range. *** = p-value <2.2e^−16^, t-test.

To generate a genome-wide view of Pc binding in wild type and tTAF (*can*; FBgn0011569) mutant testes, we performed ChIP together with microarray analysis (ChIP-array) on chromatin from whole testes. For ChIP we followed the approach we used previously to investigate Pc binding in embryos and imaginal discs [19] using a *Pc-GFP* fly line and anti-GFP antibody. For both wild type and homozygous *can* mutant testes, we find robust Pc binding at many known Pc target genes; e.g. the bithorax complex, the Antennapedia complex and the *engrailed* (*en*; FBgn0000577) locus (Figure 1A). The Pc binding profiles at the three tTAF-dependent spermatogenesis genes *dj* (FBgn0019828), *fzo* (FBgn0011596) and *Mst87F* (FBgn0002862) that were previously assayed by Chen et al. [16], [18] are shown in Figure 1B. Although Chen et al. initially reported more than 50-fold enhanced Pc binding at *Mst87F* in *can* mutant versus wild type testes [16] this was subsequently revised, using a different normalisation strategy, to a 3-fold effect at *Mst87F* and ∼2-fold effects at *dj* and *fzo* [18]. In contrast to the Chen et al. data, we do not find evidence for enhanced Pc binding at the promoters of these genes in the *can* mutant testes; neither in the ChIP-array binding profiles (Figure 1B) nor in the average ChIP enrichment calculated for a 1 kb window centred on the transcription start sites (TSS) of these genes (Figure 1C).

Visualising the Pc binding at *dj*, *fzo* and *Mst87F* in a genomic context reveals a potential problem with the interpretation of ChIP enrichment at these loci. The small *fzo* transcription unit lies within an intron of the much larger *cnc* (FBgn0262975) gene and there is an extensive Pc binding domain that covers most of the *cnc* gene. It is thus difficult to know whether ChIP enrichment close to the TSS of *fzo* is functionally related to the regulation of *fzo* or *cnc*. A similar situation occurs at *dj*, which is associated with the Pc binding domain involved in the regulation of the Antennapedia complex.

For a more global view of Pc binding at tTAF-dependent spermatogenesis genes, we used a conservative set of 942 genes identified as genes with >4-fold down-regulation in tTAF (*sa*; FBgn0002842) mutant testes [18], and compared Pc enrichment at their TSSs with all other genes in the *Drosophila* genome. Also included in the analysis is a set of canonical Pc target genes from the embryo data of Kwong et al. [19]. For each gene we plotted the TSS Pc enrichment in *can* mutant versus wild type testes (Figure 1D). We find that the tTAF-dependent spermatogenesis genes do not cluster separately from other genes in the genome; i.e. any effect of the *can* mutation on Pc binding in the spermatogenesis gene set is not dramatic. In contrast, the canonical Pc targets cluster distinctly at high enrichment values. For each gene set we also plotted the enrichment distributions (Figure 1E). The tTAF-dependent spermatogenesis gene TSSs show little evidence of Pc binding; for wild type, the median log_2_ enrichment = 0.17 (1.13 fold enrichment), and for *can* mutant, the median log_2_ enrichment = 0.47 (1.39 fold enrichment). These small positive enrichment values are similar to the enrichments seen for a set of housekeeping genes, which are expressed independently of tTAFs (see Materials and Methods for the definition of this set). In comparison, the canonical Pc targets show considerably higher enrichment; for Pc binding at the TSSs, the wild type median log_2_ enrichment = 0.90 (1.87 fold enrichment) and the *can* mutant median log_2_ enrichment = 0.73 (1.66 fold enrichment), whereas for Pc binding at a set of top embryo Pc peaks (see Materials and Methods for the definition of this set), the wild type median log_2_ enrichment = 1.65 (3.14 fold enrichment) and the *can* mutant median log_2_ enrichment = 1.10 (2.15 fold enrichment). The level of Pc binding at the spermatogenesis genes is similar in wild type and *can* mutant testes, although there is a slightly enhanced level of Pc binding in the *can* mutant testes (the *can*/wild type enrichment ratio is 1.23; p-value = 2.2e^−16^).

In summary, our data does not replicate the specific effects seen by Chen et al. at *dj*, *fzo* and *Mst87F*, and the genome-wide analysis indicates little evidence for a biologically meaningful effect of the tTAF mutation on Pc binding in the tTAF-dependent spermatogenesis gene set. We ascribe the small differences in Pc binding between wild type and tTAF mutant (at both spermatogenesis genes and canonical Pc targets) to technical difficulties in comparing the two different ChIP samples. Wild type and mutant testes differ in several ways and may not be directly comparable; *can* mutant testes are smaller in size and lack post-meiotic cells including mature sperm, have an expanded primary spermatocyte population, contain many dead and dying cells [20] and likely differ from wild type in the relative proportions of germline and somatic cells. Furthermore cellular heterogeneity makes the interpretation of data derived from whole testis analysis generally problematic as it is unclear in which cell type a particular binding event occurs.

### Pc binding in purified germline precursors

**Figure 2:**
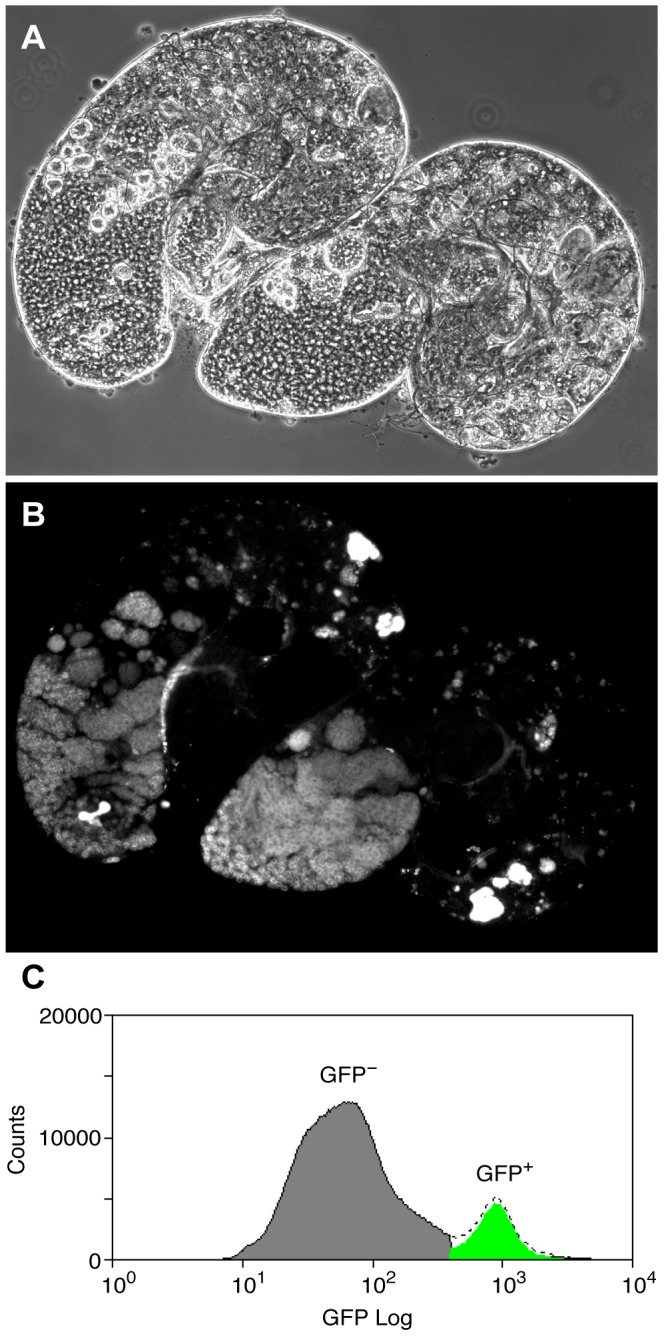
The germline precursor population. (A,B) *bam* mutant *vas-GFP* testes; the phase contrast image (A) shows the expanded precursor population and lack of primary spermatocytes and later stages; the fluorescence image (B) shows the GFP^+^ precursor cells. (C) FACS histogram showing the sorted precursor cells in green. The GFP-negative population (grey) represents somatic cells and debris.

To overcome the problem of cellular heterogeneity and more directly test the Chen et al. model, we decided to investigate Pc binding in a cell-defined manner, in germline precursors where Pc is expected to repress the tTAF-dependent spermatogenesis genes. To specifically identify precursor cells we used homozygous *bam* (FBgn0000158) mutant flies expressing the germline marker *vas-GFP*. In *bam* mutant testes, spermatogonia are prevented from differentiating into primary spermatocytes [21], [22] and so the Vas-GFP^+^ cells in *bam* mutant testes represent the germline precursor population of stem cells and spermatogonia (Figure 2). The primary spermatocyte transcription program is not activated in *bam* mutant testes; 76% of the tTAF-dependent spermatogenesis gene set has RPKM<1 [23]. For ChIP-array on the precursor population, cells were released from dissected testes, fixed, sorted on a Fluorescence-Activated Cell Sorter (FACS) according to specific GFP signals (Figure 2C and Figure S2) and chromatin was prepared from the sorted cells and immunoprecipitated using anti-Pc antibody.

**Figure 3:**
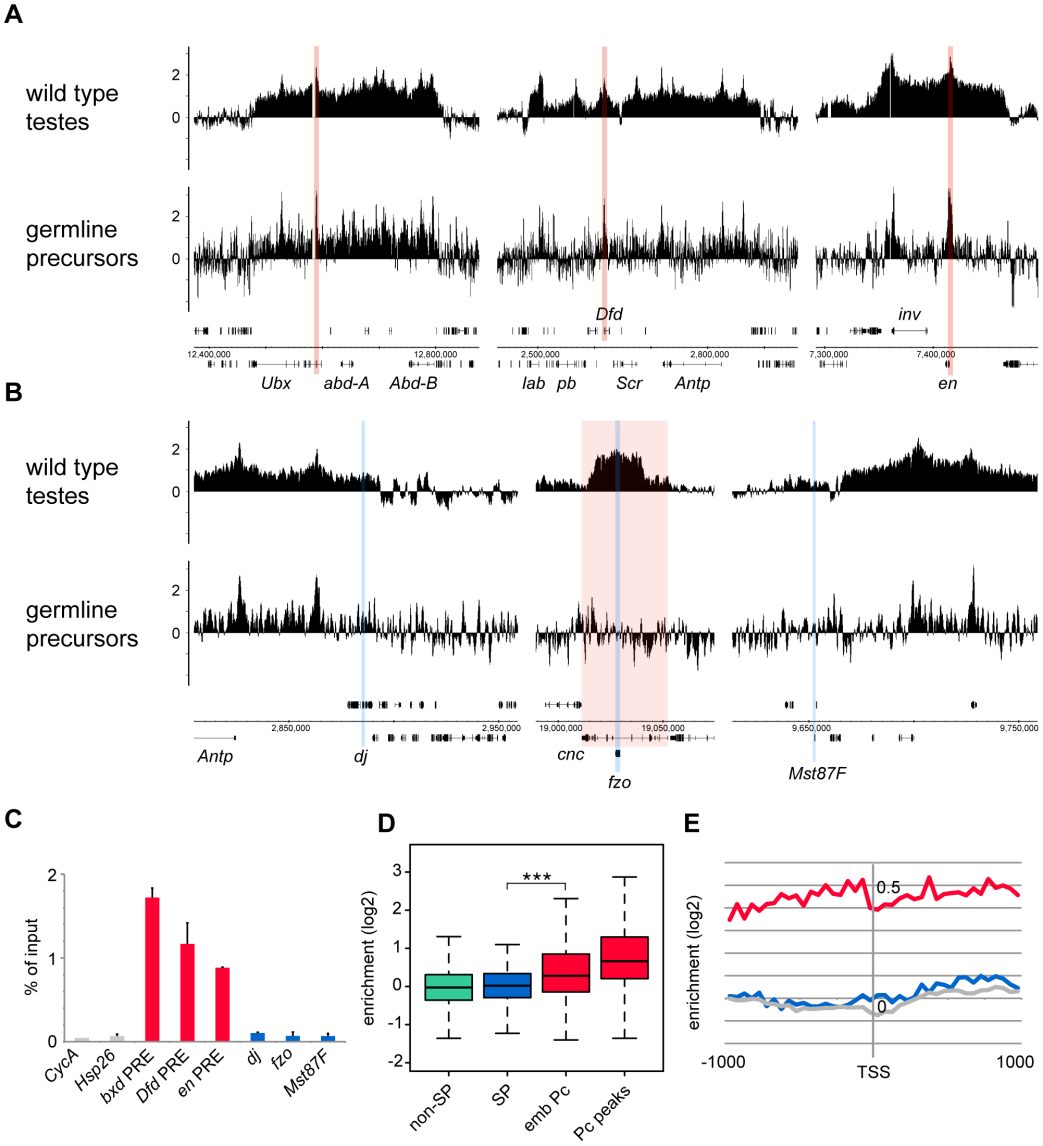
Pc binding in the purified germline precursor population. (A,B) Log_2_ ChIP enrichment profiles for Pc in germline precursors, with wild type whole testis data shown for comparison. Profiles are shown for the canonical Pc targets of the bithorax complex, the Antennapedia complex and the *en* locus (A; the *bxd*, *Dfd* and *en* PREs are indicated by red vertical stripes), and for the three tTAF-dependent spermatogenesis genes *dj*, *fzo*, and *Mst87F* (B; blue vertical stripes, red shading indicates the extent of the *cnc* gene). Linear scaled ChIP enrichment profiles are presented in Figure S1. (C) Quantitative PCR analysis of Pc enrichment in purified germline precursors at three canonical Pc targets (red), three tTAF-dependent spermatogenesis genes (blue) and two control genes (grey) having tTAF-independent regulation (error bars = 1 SD from 2 independent biological replicates, enrichment at *CycA* (FBgn0000404) is from one biological replicate). (D) Box plots showing the distribution of TSS Pc enrichments for defined gene sets in germline precursors; tTAF-dependent spermatogenesis genes (SP, blue), embryo Pc targets (emb Pc, red), top embryo Pc peaks (Pc peaks, red), and non-spermatogenesis genes (non-SP, green). Boxplots show median, 25^th^ and 75^th^ percentiles and 1.5× interquartile range. *** = p-value <2.2e^−16^, t-test. (E) Accumulated log_2_ ChIP enrichment profiles for Pc at the TSS of tTAF-dependent spermatogenesis genes (blue), embryo Pc targets (red) and random genes (grey) in germline precursors.

Although the germline precursors show clear Pc binding at many known Pc targets; e.g. the *bxd* Polycomb Response Element (PRE) in the bithorax complex, the *Dfd* PRE in the *Antennapedia* complex and the *en* PRE (Figure 3A), the binding profiles show little evidence of Pc binding at the three selected tTAF-dependent spermatogenesis genes *dj*, *fzo* and *Mst87F* (Figure 3B). The situation at *fzo* is interesting with regards to the extensive Pc domain that is associated with the overlapping *cnc* gene in whole testes. Whereas in whole testes, the *cnc* gene is strongly bound by Pc, in the precursors there is very little Pc bound to this region. This fits with RNA-seq data from *bam* mutant whole testes reporting that the *cnc* gene is likely to be expressed in precursor cells; *cnc* is expressed at a 2.4-fold higher level in precursor-enriched *bam* mutant testes compared to wild type testes [23]. Analysis of the GFP-negative FACS population indicates that Pc binding at *cnc* may represent signal coming from somatic cells (Figure S3). This largely removes the complication of *cnc*-domain Pc binding for the analysis of binding specifically at *fzo*, and reveals no clear Pc binding associated with the *fzo* gene.

Quantitative PCR analysis confirms these microarray results (Figure 3C). There is clear Pc binding at selected canonical Pc targets (the *bxd*, *Dfd* and *en* PREs), but no evidence for Pc binding at the three selected spermatogenesis genes.

Analysis over the entire set of tTAF-dependent spermatogenesis genes provides a broader view that supports these results (Figure 3D). There is no evidence for Pc binding at the spermatogenesis genes (median log_2_ enrichment = 0.02 (1.01 fold enrichment)) and no significant difference in the binding at spermatogenesis genes versus non-spermatogenesis genes. The canonical Pc target genes on the other hand show higher enrichment, assayed at either the TSS (median log_2_ enrichment = 0.28 (1.21 fold enrichment)) or at a set of top embryo Pc peaks (median log_2_ enrichment = 0.66 (1.58 fold enrichment)) although compared to whole testes the median enrichments are modest. We suggest that this is, at least in part, due to differences in Pc targets between germline precursors and the embryo. Accumulated binding profiles at the TSS support the lack of Pc binding at tTAF-dependent spermatogenesis genes, showing clear Pc binding at the canonical Pc targets, but Pc binding at the spermatogenesis genes at a background level similar to that for random gene sets (Figure 3E).

In addition, we tested whether the tTAF-dependent spermatogenesis genes that showed most enhanced Pc binding in *can* mutant versus wild type testes were preferentially associated with Pc binding in precursors. We found that the top 200 genes, having the highest *can*/wild type Pc enrichment ratios, show no Pc enrichment in precursors (median log_2_ enrichment = 0.03 (1.02 fold enrichment)), and overall we see no correlation between *can*/wild type enrichment ratio and enrichment level in precursors (Pearson correlation = 0.07).

In summary, these results from our cell-defined analysis of Pc binding do not support the Chen et al. model, which predicts that Pc should be present at the tTAF-dependent spermatogenesis genes in the germline precursors.

### Sub-nuclear localisation of Pc and tTAFs in primary spermatocytes

**Figure 4:**
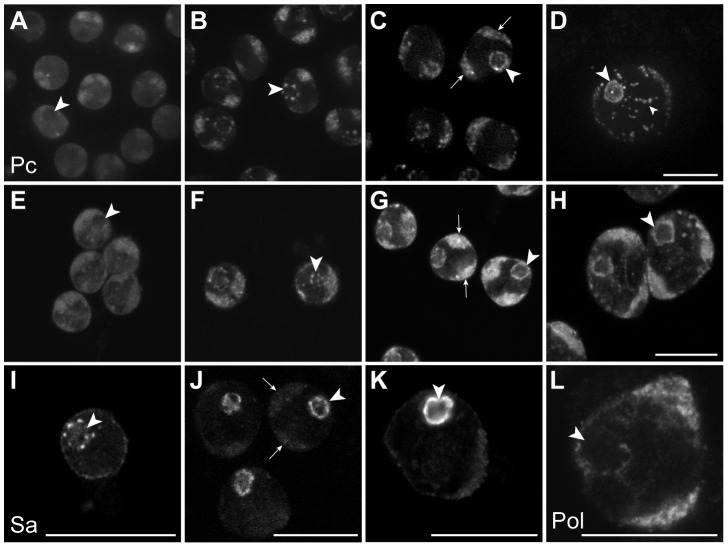
Sub-nuclear localisation of Pc, the tTAF Sa and elongating RNA polymerase II in primary spermatocytes. (A–H) Pc-GFP in live (A–D) and fixed immunolabelled (E–H) primary spermatocytes showing representative z sections of very early (A,E), early (B,F), mid (C,G) and mature (D,H) spermatocytes. (I–K) Sa-GFP in fixed immunolabelled primary spermatocytes showing representative z sections of early (I), mid (J) and mature (K) spermatocytes; a similar localisation is seen in live primary spermatocytes (data not shown). (L) Elongating RNA polymerase II (phosphoserine 2) in a mature primary spermatocyte nucleus. Large arrowheads indicate nucleolus; arrows indicate autosomal chromatin masses. The nucleoplasmic puncta (small arrowhead) in D are associated with Y loops and are not present in XO testes (data not shown). Scale bars represent 16 µm; A–D and E–H are at the same magnification.

In the Chen et al. model, the tTAF-dependent displacement of Pc from spermatogenesis gene promoters in primary spermatocytes is accompanied by a tTAF-dependent sequestration of Pc to a compartment inside the nucleolus. This sequestration is thought to indirectly aid Pc displacement by reducing the nucleoplasmic concentration of Pc available for binding. The key findings were that Pc and tTAFs both colocalise to the nucleolus in primary spermatocytes, Pc localisation coincides with tTAF expression, and Pc fails to localise to the nucleolus in tTAF mutants [16]. Given that our cell-defined ChIP-array analysis provides no evidence for the repressive role of Pc in spermatogenesis as proposed in the Chen et al. model, we decided to re-examine the localisation of Pc and tTAFs in primary spermatocytes. In live *Pc-GFP* testes, in very early primary spermatocytes we find Pc present on the chromosome masses, diffusely present in the nucleoplasm and excluded from the nucleolus (Figure 4). Later, in addition to remaining associated with the chromosome masses, Pc accumulates in clumps at the periphery of the nucleolus and subsequently coats the nucleolar surface appearing as a ring around the nucleolus. Localisation of Pc in fixed testes shows a similar pattern to that in live testes. The localisation of the tTAF Sa, in both live and fixed *Sa-GFP* testes, is similar to that of Pc although Sa is not expressed in very early primary spermatocytes. It appears weakly associated with the chromosome masses and more strongly with the nucleolus, initially accumulating in clumps at the nucleolar periphery before forming a ring around the nucleolus. Consistent with previous findings [16], we conclude that the timing of Pc localisation to the nucleolus coincides with the expression of Sa in early primary spermatocytes. However our data does not agree with Chen et al.'s observation that Pc is sequestered inside the nucleolus as, although we see some label within the nucleolus, the predominant Pc labelling is at the nucleolar periphery. Since elongating RNA polymerase II is also present at the nucleolar periphery (Figure 4L) it seems that Pc, rather than being sequestered and inactive, is localised to a region where it may directly participate in gene regulation. We still lack an understanding of why tTAFs show such a striking concentration at the nucleolar periphery and one possibility is that this might be the major site of spermatogenesis gene transcription. We tested this possibility by examining the sites of transcription of two spermatogenesis genes, *fzo* (a tTAF-dependent gene) and *bol* (FBgn0011206; a tTAF-independent tMAC-dependent gene), in primary spermatocyte nuclei. Although we find evidence of transcribed genes looping out a short distance from their chromosome territories, neither gene was transcribed at the nucleolar periphery (Figure S4). A more comprehensive analysis will be needed to resolve this issue.

## Discussion

We have used genome-wide and cell-specific analysis to investigate the role of Pc in the regulation of the spermatogenesis transcription program in primary spermatocytes. In the current model of spermatogenesis gene activation [16], spermatogenesis genes are repressed in the precursor cells by the PcG silencing machinery and are switched on in primary spermatocytes by tTAF-dependent displacement of Pc. The model was based on ChIP results from whole testes with the principal observation being that Pc binding at tTAF-dependent spermatogenesis genes is enhanced in tTAF mutant testes compared to wild type. Our genome-wide whole-testis analysis however shows no evidence for any substantial enhancement in Pc binding in tTAF mutant testes, neither at selected spermatogenesis genes nor across the tTAF-dependent spermatogenesis gene set as a whole. We consider that the slight enhancement we observe is more likely to reflect technical difficulties in comparing wild type and mutant testes, than to reflect a real biological mechanism. In support of this, our cell-defined analysis of purified germline precursors shows no clear evidence of Pc binding at the spermatogenesis genes. Overall, our data argues against a model of transcription switch-on based on the release of Pc.

If the switch-on of the spermatogenesis genes in primary spermatocytes is not mediated by the release of Pc silencing, how then does it occur? A recent study in *Drosophila* has revealed a role for B-type lamins in the repression of large testis-specific gene clusters in somatic cells [24]. These gene clusters are highly enriched for lamin binding in somatic cells but are not enriched for Pc binding. The genes contained within these clusters are up-regulated in lamin B RNAi-treated tissue culture cells, and in somatic larval tissue homozygous for a null lamin B allele. Also, in the testes, onset of spermatogenesis gene transcription in primary spermatocytes is associated with release of these genes from the nuclear lamina. In the analysis of chromatin domain types in the *Drosophila* Kc somatic cell line, lamin is predominantly associated with BLACK chromatin whereas Pc is a signature of BLUE chromatin [25]. Using this chromatin domain data we find that the tTAF-dependent spermatogenesis genes are more prominently enriched in BLACK chromatin (1.9-fold enrichment) than in BLUE (1.3-fold enrichment), emphasising the potential importance of release from lamina association in the switch-on of the primary spermatocyte transcription program (Figure S5).

A curious feature of Pc localisation in primary spermatocytes is its association with the nucleolus [26]. Although this has been suggested to represent sequestration of Pc [16], here we argue that the concentration of Pc at the periphery of the nucleolus (rather than inside it) is more consistent with an active role for Pc in gene regulation. Intriguingly, tTAFs are also associated with the nucleolus [16] and, similarly to Pc, are concentrated at the nucleolar periphery (this study, and see [27] for TAF1). The functional relevance of Pc and tTAF localisation at the nucleolus is unclear. We have investigated the possibility that autosomal spermatogenesis genes might be transcribed at the periphery of the nucleolus but a limited sampling of two genes provided no evidence for this (Figure S4). Another possibility is that the nucleolar-localised Pc and tTAFs are involved in regulation of the sex chromosomes (X and Y) associated with the nucleolus. An X chromosome connection is suggested by the observation that TAF1 nucleolar localisation is not dependent on the presence of the Y chromosome [27]. In this regard it is interesting that during spermatogenesis there is evidence for a yet uncharacterized mechanism of X chromosome-specific repression; X-linked transgenes are expressed at a much lower level than autosomal transgenes and the magnitude of this expression difference is too large to be explained by the lack of hypertranscriptional X chromosome dosage compensation in primary spermatocytes [28]. It is thus possible that Pc and tTAFs at the periphery of the nucleolus may reflect some aspect of this X chromosome-specific regulation.

Although for a long time the PcG silencing machinery (including both Polycomb Repressive Complex 1 and 2; PRC1 and PRC2) has been associated with the permanent heritable silencing of the Hox genes, it has more recently been shown to have a dynamic role in cell fate decisions and the regulation of lineage-specific gene expression programs (reviewed in [17], [29]). Studies on ES cell differentiation have provided a powerful paradigm for the role of PcG complexes in developmental decisions. In the undifferentiated state, key lineage-specific developmental control genes are expressed at only very low levels and are associated with a poised state of gene activation, with gene activators being balanced by the repressive effect of the PcG silencing machinery [30]. This poised state is characterised by bivalent chromatin harbouring both repressive (H3K27me3) and active (H3K4me3) marks and is associated with paused RNA polymerase II [30]–[33]. Lineage-specific signals induce differentiation to a specific cell fate, and for example for a neural cell fate, this is accompanied by the specific loss of the PcG machinery and associated repressive histone marks from neural-lineage genes resulting in their stable expression [34], [35]. Similar mechanisms have been shown to operate in other stem cell lineages (e.g. myogenesis [36] and haematopoiesis [37]) and the association of PcG complexes with a wide range of developmental regulators suggests that PcG silencing is pervasively involved in developmental cell fate decisions [38]–[41]. In contrast, our data argues against a role for Pc in the regulation of the primary spermatocyte gene expression program. It remains unclear as to whether any of the other PcG proteins are involved in regulating the primary spermatocyte gene expression program, or whether the PcG machinery regulates gene expression elsewhere in the spermatogenesis differentiation pathway. However, a lack of requirement for Pc may reflect a germline/soma difference as Pc is not necessary for normal oogenesis [42].

The activation of the spermatogenesis transcription program in primary spermatocytes has features that may set it apart from the ES cell differentiation paradigm. Firstly, it is dependent on tissue-specific core transcriptional machinery, involving at least five testis-specific TAF components (reviewed in [1]). This suggests that in the absence of these testis-specific core components the spermatogenesis genes are unrecognized by RNA polymerase II, preinitiation complex assembly fails to take place, and spermatogenesis genes are inactive. Indeed Gan et al. [43] find that spermatogenesis differentiation genes are not associated with poised RNA polymerase II (or bivalent chromatin marks) in precursor-enriched *bam* mutant testes. Most components of the tTAF and tMAC complexes and the homeodomain transcription factors, Achi and Vis, are not expressed in the precursor cells [2]–[6], [14]–[16]. Also Chen et al. [16], [18] report that inactivating components of the PcG machinery, either PRC1 or PRC2, in the absence of tTAFs is insufficient to switch on spermatogenesis gene expression in primary spermatocytes. This situation contrasts with differentiation events where transcription factors and poised RNA polymerase II are engaged at differentiation genes in undifferentiated cells and the PcG machinery is required to repress transcription to low levels to preserve the undifferentiated state or protect against aberrant or premature differentiation. Spermatogenesis genes may not require Pc-dependent silencing in precursors if there is no enhancer/promoter activity present to drive transcription. The second feature of the primary spermatocyte transcription program that is clearly distinct from the ES cell differentiation paradigm is that, whereas ES cells are pluripotent and can differentiate into a variety of different cell fates, germline precursors are unipotent destined to the single fate of differentiating into primary spermatocytes and activating the primary spermatocyte transcription program. This may require a simpler mechanism of transcriptional control. In ES cells, developmental genes representing multiple differentiation pathways are poised for activation with the engagement of sets of transcription factors and paused RNA polymerase II, and in this context PcG silencing is required to hold ES cells in a plastic multi-potential paused state in anticipated response to any cell differentiation cue. In contrast, germline precursors have no alternative pathways; switching on the spermatogenesis genes may only require the activation of a small set of transcriptional regulators and core transcriptional components.

## Materials and Methods

### Fly stocks

For sorting GFP^+^ germline precursor cells, we used double homozygous *vas-GFP; bam^Δ86^* flies from *vas-GFP; bam^Δ86^*/TM3 stocks generated using transgenic *w; vas-GFP* (DGRC Kyoto stock center, [44]) and *bam^Δ86^*/TM3 (Bloomington stock center, [45]). For the analysis of Pc in wild type whole testes, we used homozygous transgenic *w; ; Pc-GFP* [26] flies. For the analysis of Pc in tTAF mutant whole testes, we used double homozygous *Pc-GFP; can^3^* flies from *Pc-GFP; can^3^*/TM3 stocks generated using transgenic *w; Pc-GFP* [26] and *can^3^*/TM3 [20] flies. For immunolabelling, we used transgenic *Pc-GFP* [26] and *Sa-GFP* [16] flies.

### Testis dissections, cell extraction and fixation

Testes were dissected in ice-cold Schneider's medium (supplemented with 10% fetal calf serum) and incubated with collagenase (5 mg ml^−1^, Sigma-Aldrich C8051) plus protease inhibitors (Sigma-Aldrich P8340) in medium for 5 min at room temperature. After washing in PBS, cells were extracted by vigorously pipetting for 1 min, using a narrow-ended P200 tip (Rainin RT-200F), in 100 µl PBS; and fixed by adding an equal volume of 2% formaldehyde (Sigma-Aldrich F8775) in medium, mixing thoroughly, and incubating for 15 min at 37°C in an Eppendorf Thermomixer at 700 rpm. Fixation was stopped by adding 400 µl ice-cold medium and placing the sample on ice. The sample was spun down in a swing-out rotor at 1,000 *g* for 5 min at 4°C, and the pellet snap frozen in liquid N_2_ and stored at −80°C. Approximately 1,000 testes were dissected for each ChIP-array replicate. Testes were dissected in batches of 70, and for each batch, the time lapsing from the start of dissection till fixation was approximately 1 hr.

### FACS

Aliquots of extracted cells stored at −80°C were thawed, combined in PBS/0.01% Triton X-100 and 50 µm filtered (Partec 04-004-2327). Cells were sorted using a 100 µm nozzle on a MoFlo FACS machine (Beckman Coulter) equipped with a 488 nm argon laser (100 mW). Cells were sorted into a microfuge tube containing 700 µl PBS/0.01% Triton X-100. Events were triggered on forward scatter and GFP^+^ events were sorted using the gating strategy described in Figure S2. Data was acquired and analysed using Summit software (Beckman Coulter).

### ChIP on sorted cells

Sorted cells were spun down in a swing-out rotor at 4,000 *g* for 15 min at 4°C, transferred to a thin-walled 0.5 ml microfuge tube (Axygen PCR-05-C), re-spun down and resuspended in 130 µl Lysis Buffer (17 mM Tris.HCl (pH 8), 3.4 mM EDTA.Na_2_, 0.34% SDS) containing protease inhibitors (Sigma-Aldrich P8340), and sonicated for 5 cycles at high setting using a Diagenode Bioruptor (1 cycle is 30 s ON and 30 s OFF). After sonication, the sample was centrifuged at 16,000 *g* for 15 min at 4°C, the chromatin-containing supernatant transferred to a fresh microfuge tube, and 70 µl RIPA buffer (36.7 mM Tris.HCl (pH 8), 2.5 mM EDTA.Na_2_, 0.01% SDS, 2.46% Triton X-100, 374 mM NaCl) containing protease inhibitors added to the chromatin sample. The ChIP reaction, washes and DNA purification were performed as in Dahl and Collas [46], [47]. In brief, magnetic beads were coated with 1 µl of rabbit anti-Pc antibody [48], and incubated overnight with 180 µl of chromatin (equivalent to ∼85,000 GFP^+^ sorted events). Beads were washed, chromatin eluted, RNA and proteins digested, and the DNA then purified by phenol/chloroform extraction and ethanol precipitation using linear acrylamide as carrier and resuspended in 10 µl PCR grade water. Approximately 10 µl of chromatin was retained as input and purified alongside the ChIP sample. Quantitative PCR primers used for Pc enrichment analysis are listed in Table S1.

### ChIP on whole testes

A total of 100 testes were dissected from wild type *Pc-GFP* or *can* mutant *Pc-GFP* flies and fixed using 1% formaldehyde in PBS for 15 min at 37°C. Fixation was stopped by washing in PBS/125 mM Glycine/0.01% Triton X-100. Chromatin preparation was performed as described above, except that before sonication testes were homogenized using a motor-driven plastic pestle (Sigma-Aldrich Z359947) in a thin-walled 0.5 ml tube containing 130 µl Lysis Buffer. The ChIP reaction, washes and DNA purification were performed as described above. The ChIP reaction used 1 µl of rabbit anti-GFP antibody [49]. Approximately 10 µl of chromatin was retained as input and purified alongside the ChIP sample.

### Amplification and labelling of ChIP DNA

ChIP and input DNA were amplified using the GenomePlex Single Cell Whole Genome Amplification Kit (Sigma-Aldrich WGA4) following the manufacturer's instructions from the library preparation stage. Approximately 150 pg of DNA was used for amplification. Samples were amplified for 22 cycles (sorted cells) or 20 cycles (whole testes), and amplified DNA was purified using the QIAquick PCR Purification Kit (Qiagen). The amplified ChIP and input DNA (1 µg for sorted cells, 2 µg for whole testes) were labelled with Cy5 and Cy3 using the NimbleGen Dual-Color DNA Labeling Kit for sorted cells, or the BioPrime DNA Labeling Kit (Invitrogen) in the presence of Cy3- or Cy5-dCTP (GE Healthcare) for whole testes, and hybridised onto Nimblegen ChIP-chip 2.1M Whole-Genome Tiling Arrays according to the manufacturer's instructions.

### Microarray data processing

We performed two biological replicates for each sample, except wild type *Pc-GFP* whole testes where we performed three. A Cy3/Cy5 dye swap was performed for one biological replicate of each sample. For all ChIP-array experiments, input chromatin was used as the reference control to assay ChIP enrichment. Arrays were scanned and the scanned images processed using NimbleScan software to generate pair files, which were then converted into chromatin enrichment profiles (log_2_ ChIP/input ratio) using the TiMAT pipeline (http://bdtnp.lbl.gov/TiMAT/). Samples were median scaled at 500, quantile normalized (ChIP and input samples together), and window smoothed using a window size of 500 bp and a minimum number of oligos per window of 4. Enrichment profiles were visually examined using the Integrated Genome Browser (http://bioviz.org/igb/index.html). The ChIP-array data has been submitted to Gene Expression Omnibus under accession number GSE39935.

### Bioinformatic analysis

All analyses are based on release 5.48 of the *Drosophila melanogaster* genome. Gene lists for spermatogenesis genes were derived from Chen et al. [18]; GEO accession number GSE28728. The tTAF-dependent spermatogenesis genes (942 genes) were identified as >4-fold down-regulated in *sa* mutant testes. The tMAC-dependent spermatogenesis genes (1,448 genes) in Figure S5 were identified as >4-fold down-regulated in *aly* (FBgn0004372) mutant testes. The embryo Pc target gene set (386 genes) is from Kwong et al. [19]. The housekeeping gene set (4,184 genes) used the FlyAtlas data [50] selecting genes expressed in all tissues (4 present calls). The non-spermatogenesis gene set includes all genes in the genome except the tTAF-dependent spermatogenesis gene set. Top embryo Pc peaks were defined as Pc binding intervals (433 intervals) with log_2_ ChIP enrichments >1.0 in Kwong et al. [19] embryo data, and for each interval Pc enrichment was calculated using a 1 kb window of ChIP oligo scores centred on the interval midpoint. Pc enrichment at the TSS was calculated using a 1 kb window of ChIP oligo scores (from −500 bp to +500 bp relative to the TSS using the 5′ end of Flybase release 5.48 gene models as TSS positions). Accumulated binding profiles were average profiles obtained using 50 bp bins. The random profile in Figure 3E is derived from the average of 10 random samplings of 942 genes from 15,147 FBgns from FlyMine (http://www.flymine.org/).

### Immunolabelling and RNA in situ hybridisation

Immunolabelling was performed as in Redhouse et al. [51]. Primary antibodies used were: rabbit anti-GFP (1∶8000, Invitrogen A6455), mouse anti-histone (1∶1000, Chemicon clone 52) and rabbit anti-RNA polymerase II phosphoserine 2 (1∶300, Abcam ab5095). Secondary antibodies used were: goat anti-mouse Alexa 488 and goat anti-rabbit Alexa 488 (1∶400, Invitrogen). RNA in situ hybridisation was performed following immunolabelling using the method described in Kosman et al. [52] with digoxigenin-labelled antisense RNA probes generated by in vitro transcription of PCR-amplified *bol* and *fzo* DNA fragments. The *bol* probe was generated from a 2,851 bp intron fragment PCR-amplified using the primers: 5′ AAACGATGGCAACAAAGGAG 3′ and 5′ TGGCACAGATACGAAGCAAG 3′. The *fzo* gene lacks introns and so the *fzo* probe was generated from a 1,264 bp exon fragment PCR-amplified using the primers: 5′ GGCCCTAAAACCCTCAACTC 3′ and 5′ TAAAACGGTGCCCAAGCTAC 3′. Sheep anti-digoxigenin primary antibody (1∶500, Roche) and goat anti-sheep Alexa 568 secondary antibody (1∶400, Invitrogen) were used for detection.

## Supporting Information

Figure S1 Linear scaled ChIP enrichment profiles. ChIP enrichment profiles for Pc in wild type and *can* mutant whole testes, with embryo data [19] shown for comparison (A,B) and for Pc in germline precursors, with wild type whole testis data shown for comparison (C,D). Profiles are shown for the canonical Pc targets of the bithorax complex, the Antennapedia complex and the *en* locus (A,C; in C the *bxd*, *Dfd* and *en* PREs are indicated by red vertical stripes), and for the three tTAF-dependent spermatogenesis genes *dj*, *fzo*, and *Mst87F* (B,D; blue vertical stripes, red shading indicates the extent of the *cnc* gene).
(TIF)

Figure S2 FACS gating strategy used to sort the germline precursor population. (A–C) Gates R1, R2 and R3 were used to sort GFP^+^ germline precursors with A showing all events, B showing events after gating by R1 and C showing events after gating by both R1 and R2. Autofluorescence induced by the 488 nm laser is plotted against GFP fluorescence in order to discriminate between genuine GFP^+^ cells and autofluorescent events. (D,E) Sorted germline precursors; (D) phase contrast image, (E) fluorescence image. Sort purity >99%.
(TIF)

Figure S3 Quantitative PCR analysis of Pc enrichment in purified germline precursors and GFP-negative somatic cells. Pc enrichment is shown for the promoters of the three selected tTAF-dependent spermatogenesis genes calculated as fold enrichment relative to a negative control gene (*CycA*). Note that Pc binding is observed at *fzo* in the GFP-negative somatic cells and not in the germline precursors. Error bars = 1 SD from 2 independent biological replicates (enrichment at *CycA* in the precursors is from one biological replicate).
(TIF)

Figure S4 Localisation of transcribing spermatogenesis genes. Single z sections showing RNA fluorescent in situ hybridisation for *bol* (A–C) and *fzo* (D–F) combined with histone immunolabelling in mature primary spermatocytes. (A,D) RNA in situ; (B,E) histone labelling; (C,F) merge. The asterisk marks the nucleolus. These single z sections show one or two puncta per nucleus and overall we see four puncta per nucleus corresponding to the four gene copies in these tetraploid G2 cells. Scale bars represent 8 µm.
(TIF)

Figure S5 Spermatogenesis genes are preferentially associated with BLACK chromatin. Percentage of genes associated with each of the five chromatin domain types in Kc cells from Filion et al. [25] for defined gene sets; all genes in the genome (grey), spermatogenesis genes regulated by the tMAC component Aly (light blue) and spermatogenesis genes regulated by the tTAF Sa (dark blue).
(TIF)

Table S1 Quantitative PCR primers used for Pc enrichment analysis. The primer sequences used for the *en* PRE were taken from Langlais et al. [53].
(DOCX)
