# Supplementary figures and images for: Genome-Wide and Cell-Specific Epigenetic Analysis Challenges the Role of Polycomb in Drosophila Spermatogenesis
Source: PLoS Genet. 2013 Oct 17;9(10):e1003842. doi: 10.1371/journal.pgen.1003842 (PMC3798269; doi:10.1371/journal.pgen.1003842)

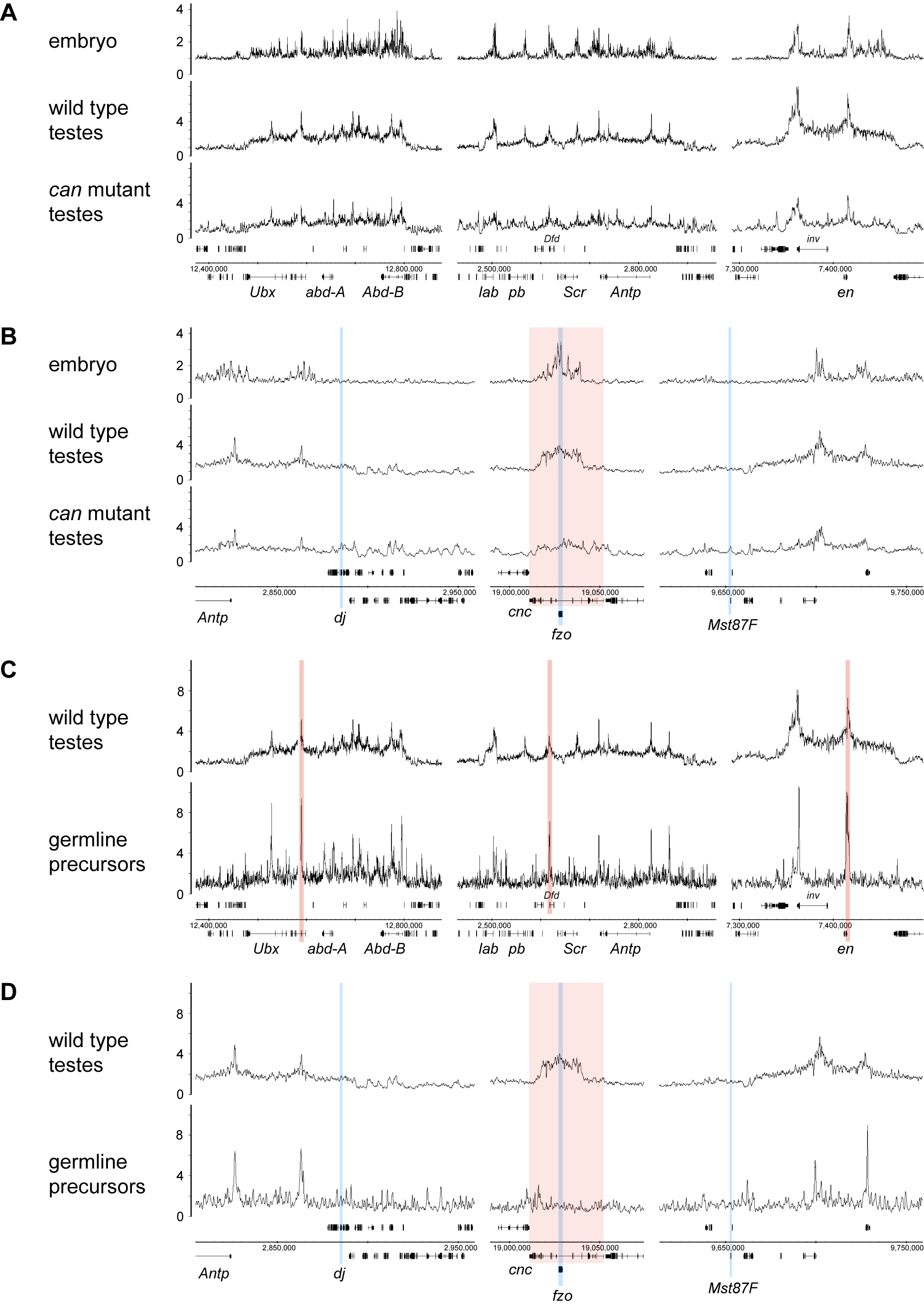

Supplement: Figure S1 — Linear scaled ChIP enrichment profiles. ChIP enrichment profiles for Pc in wild type and can mutant whole testes, with embryo data [19] shown for comparison (A,B) and for Pc in germline precursors, with wild type whole testis data shown for comparison (C,D). Profiles are shown for the canonical Pc targets of the bithorax complex, the Antennapedia complex and the en locus (A,C; in C the bxd, Dfd and en PREs are indicated by red vertical stripes), and for the three tTAF-dependent spermatogenesis genes dj, fzo, and Mst87F (B,D; blue vertical stripes, red shading indicates the extent of the cnc gene). (TIF) [file pgen.1003842.s001.tif]

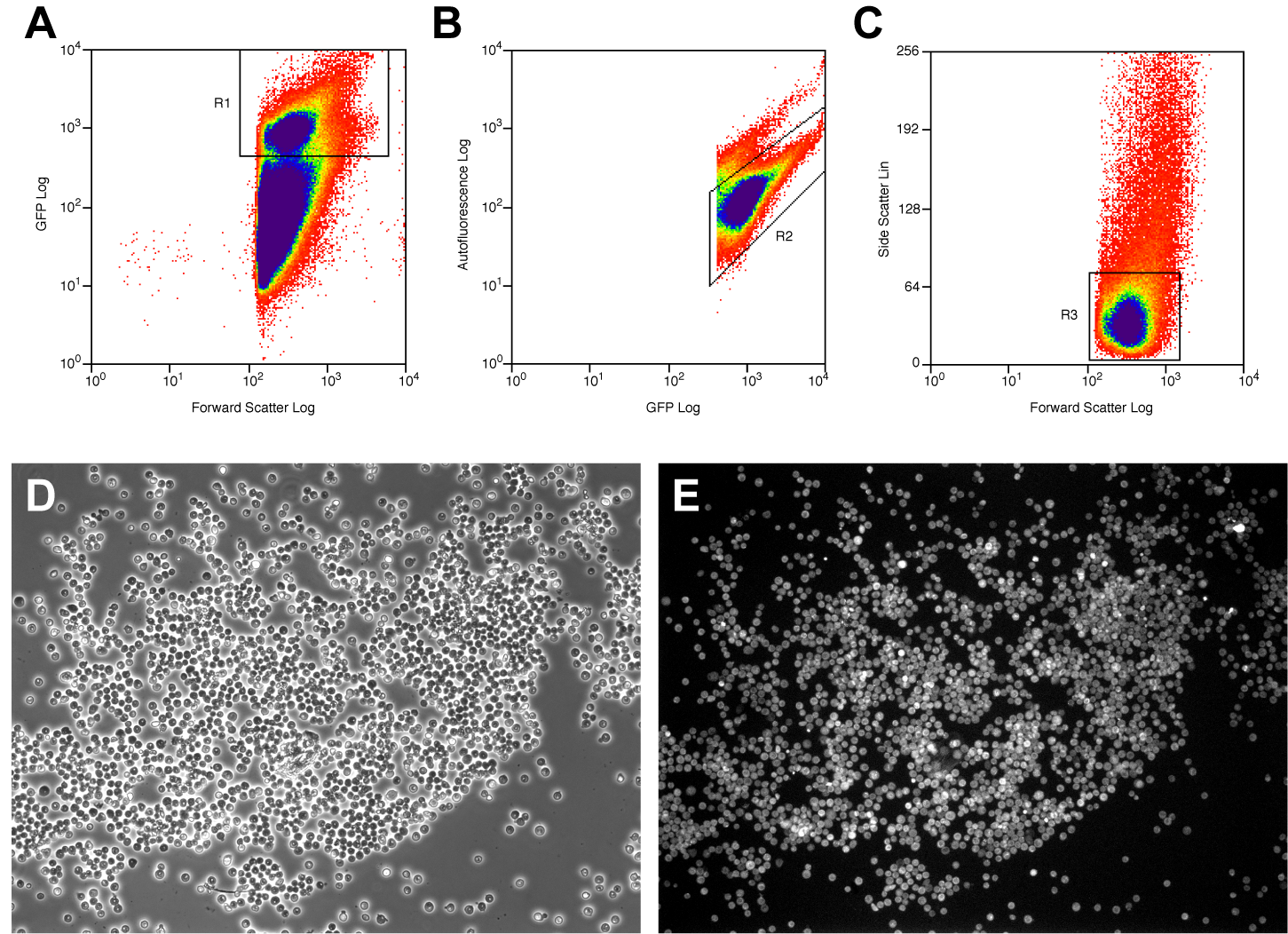

Supplement: Figure S2 — FACS gating strategy used to sort the germline precursor population. (A–C) Gates R1, R2 and R3 were used to sort GFP+ germline precursors with A showing all events, B showing events after gating by R1 and C showing events after gating by both R1 and R2. Autofluorescence induced by the 488 nm laser is plotted against GFP fluorescence in order to discriminate between genuine GFP+ cells and autofluorescent events. (D,E) Sorted germline precursors; (D) phase contrast image, (E) fluorescence image. Sort purity >99%. (TIF) [file pgen.1003842.s002.tif]

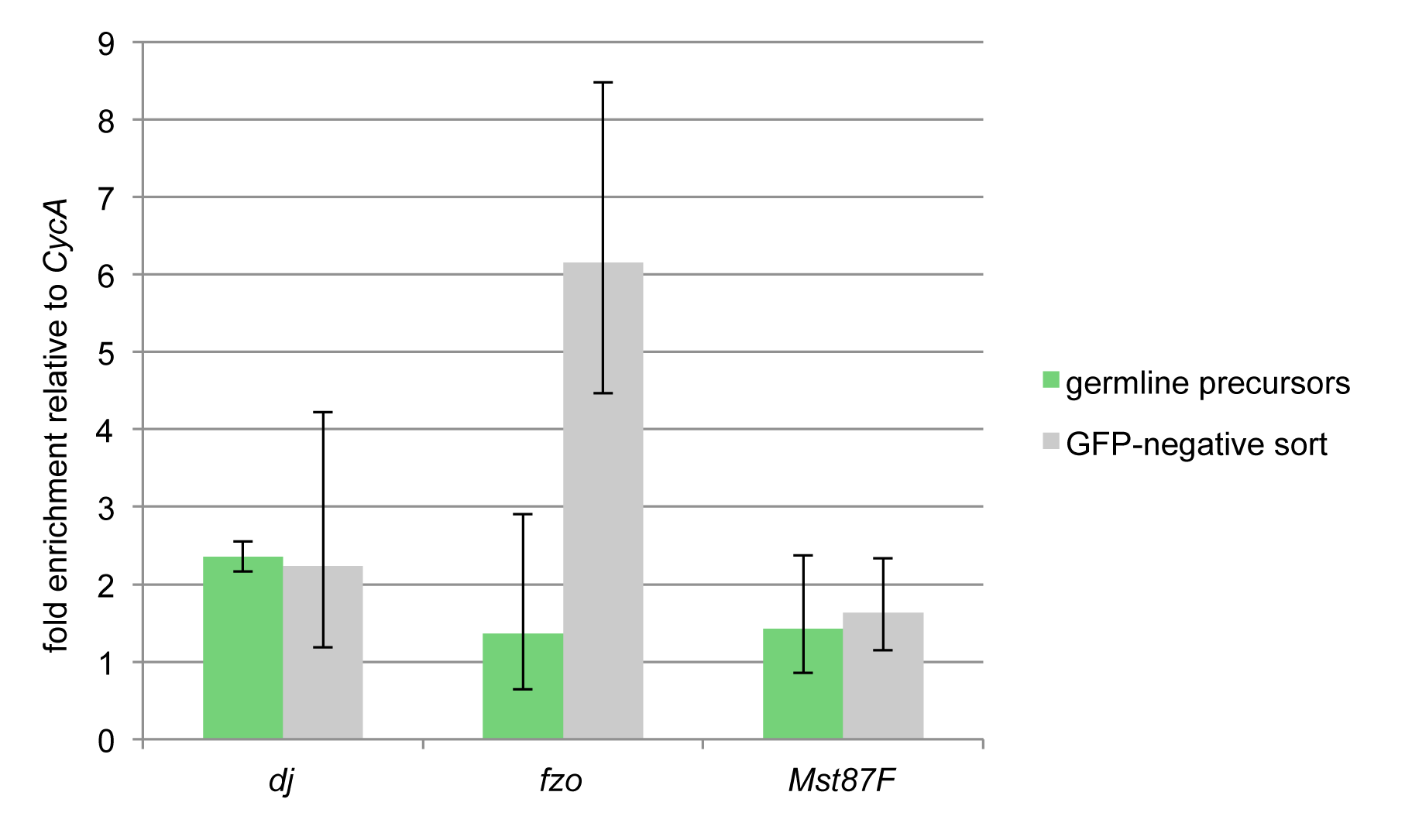

Supplement: Figure S3 — Quantitative PCR analysis of Pc enrichment in purified germline precursors and GFP-negative somatic cells. Pc enrichment is shown for the promoters of the three selected tTAF-dependent spermatogenesis genes calculated as fold enrichment relative to a negative control gene (CycA). Note that Pc binding is observed at fzo in the GFP-negative somatic cells and not in the germline precursors. Error bars = 1 SD from 2 independent biological replicates (enrichment at CycA in the precursors is from one biological replicate). (TIF) [file pgen.1003842.s003.tif]

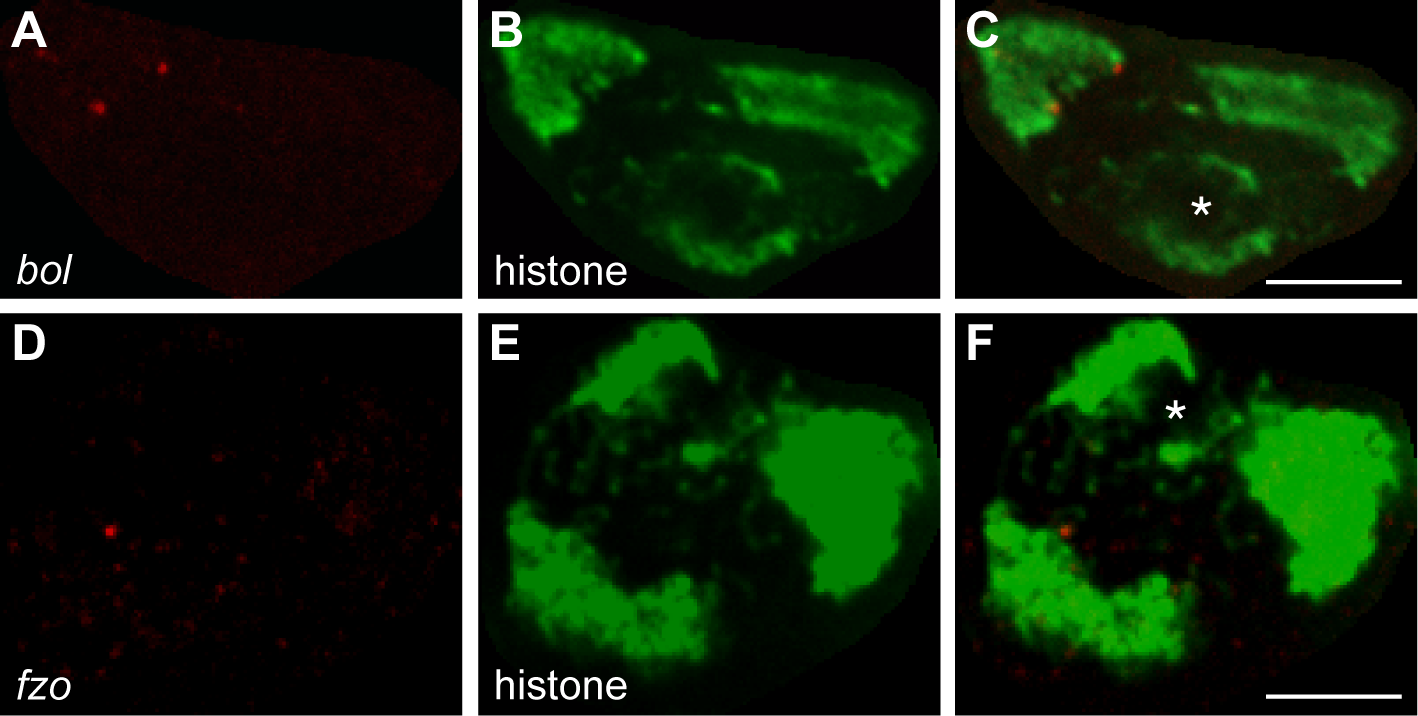

Supplement: Figure S4 — Localisation of transcribing spermatogenesis genes. Single z sections showing RNA fluorescent in situ hybridisation for bol (A–C) and fzo (D–F) combined with histone immunolabelling in mature primary spermatocytes. (A,D) RNA in situ; (B,E) histone labelling; (C,F) merge. The asterisk marks the nucleolus. These single z sections show one or two puncta per nucleus and overall we see four puncta per nucleus corresponding to the four gene copies in these tetraploid G2 cells. Scale bars represent 8 µm. (TIF) [file pgen.1003842.s004.tif]

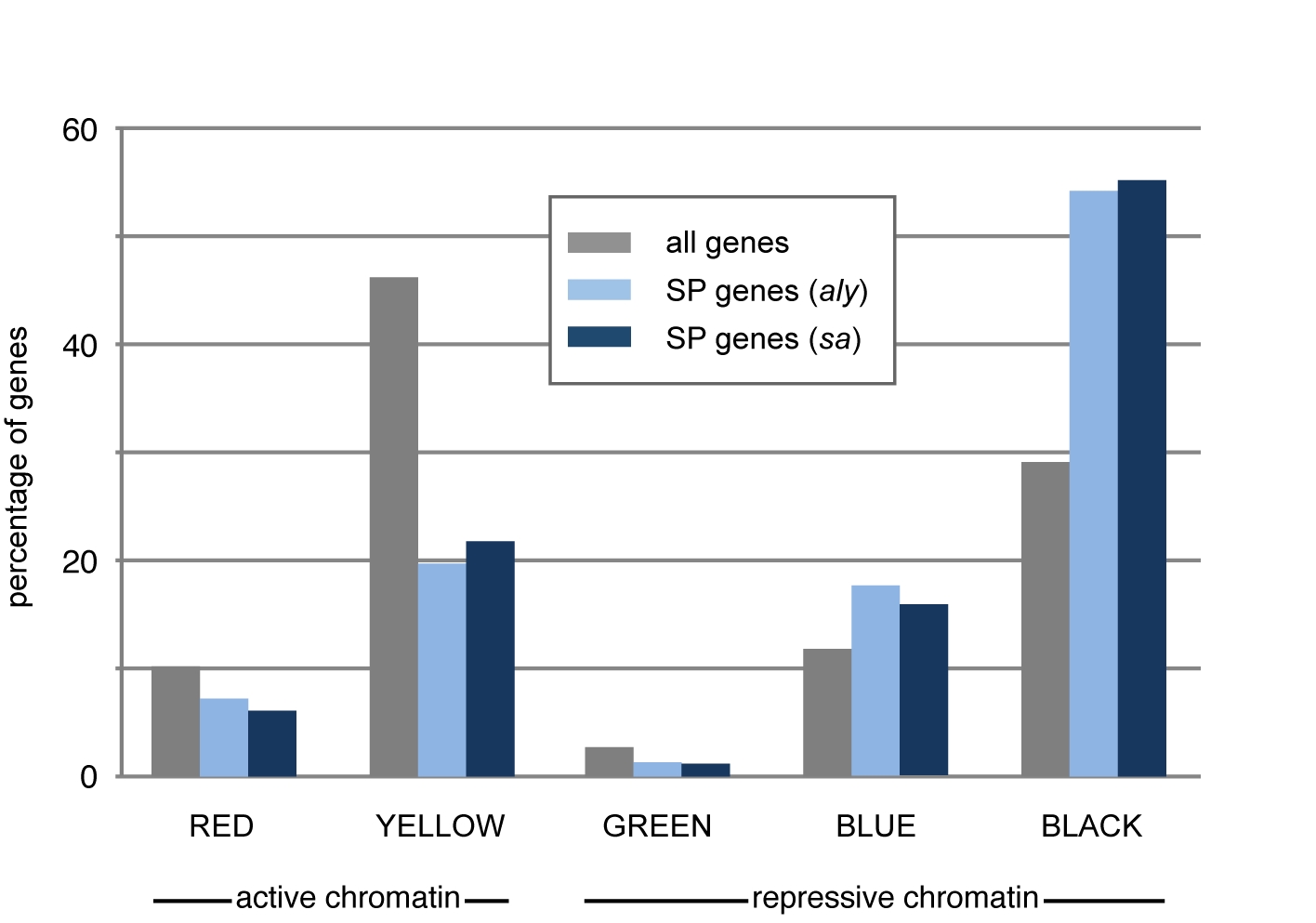

Supplement: Figure S5 — Spermatogenesis genes are preferentially associated with BLACK chromatin. Percentage of genes associated with each of the five chromatin domain types in Kc cells from Filion et al. [25] for defined gene sets; all genes in the genome (grey), spermatogenesis genes regulated by the tMAC component Aly (light blue) and spermatogenesis genes regulated by the tTAF Sa (dark blue). (TIF) [file pgen.1003842.s005.tif]
