# Supplementary material for: Genome-Wide and Cell-Specific Epigenetic Analysis Challenges the Role of Polycomb in Drosophila Spermatogenesis
Source: PLoS Genet. 2013 Oct 17;9(10):e1003842. doi: 10.1371/journal.pgen.1003842 (PMC3798269; doi:10.1371/journal.pgen.1003842)
Supplement: Table S1 — Quantitative PCR primers used for Pc enrichment analysis. The primer sequences used for the en PRE were taken from Langlais et al. [53]. (DOCX) [file pgen.1003842.s006.docx]

| *CycA* | GAAGGCAACGACGCTAAGAG |
| --- | --- |
|  | TGGTGCGCTCTTTCCTACTC |
| *Hsp26* | CGGCTCTCTCACTCATACAGG |
|  | AACTAACCTTTCCCAATAAATGC |
| *bxd* PRE | TGCGAAATGCTACTGCTCTC |
|  | TTTGAGTTATCGGCACTTTGG |
| *Dfd* PRE | ACAATGCTCCCTCTCAGTCG |
|  | TCACCTTTTCTCACCCTTCG |
| *en* PRE | TTTGGCTCCGTTCCAGTTTCCAG |
|  | TCTTAACCGGTTCGAACGCACCTT |
| *dj* | GATCCTGATTCCACAGACAAATAG |
|  | GCGGTTCTCTTAAACATTTCG |
| *fzo* | GCGAGCAAAACAACATCTACAG |
|  | ACGACGTACTTTCTCCGGAG |
| *Mst87F* | ATCCTTTGCCTCTTCAGTCC |
|  | AATAATGATACAAAATCTGGTTACGC |
